# Percutaneous real-time ultrasound-guided renal biopsy performed solely by nephrologists: A case series

**DOI:** 10.4103/0971-4065.70844

**Published:** 2010-07

**Authors:** S. S. Yesudas, N. K. Georgy, S. Manickam, A. Raheena, R. C. Monai, B. A. Noble, A. Pillai

**Affiliations:** Department of Nephrology, PVS Memorial Hospital, Kalloor, India; 1 Lakeshore Hospital and Research Centre, Nettur PO, Kochi, Kerala, India

**Keywords:** Interventional nephrology, percutaneous renal biopsy, ultrasound-guided renal biopsy

## Abstract

Renal biopsy is an integral part of the nephrologists’ diagnostic armamentarium. Usually it is performed by radiologists or nephrologists with radiologist’s assistance. Our aim was to assess the efficacy and safety of percutaneous ultrasound-guided renal biopsy performed solely by nephrologists. We performed real-time ultrasound-guided renal biopsy on 37 patients (N group). The results were then compared with those of a similar number of biopsies done with radiologist’s support (NR group) immediately prior to these. In the N group, 36 biopsies (97.3%) were successful and were histopathologically adequate, whereas in the NR group, all biopsies were successful but only 28 were adequate (75.68%). Eighteen patients required only a single attempt in the N group, whereas majority (34 patients) in the NR group required two or more attempts. The average attempt per bit of renal tissue was 1.22 in both the groups. The average number of passes per patient was 1.77 in the N group and 2.32 in the NR group. The mean size of renal tissue obtained was 1.41 ± 0.47 cm in the N group and 1.19 ± 0.42 cm in the NR group. The average number of glomeruli was 15.62 ± 5.26 and 13.7 ± 7.38 in the N and NR groups, respectively (*P*<0.05). In the N group, there were no complications except two cases of post procedural hematuria that was managed conservatively. There was no need for blood transfusion and both of them were discharged after 48 hours. No patient had peri-renal collection or hematoma on repeat ultrasonography of the abdomen at 24 hours. However, in the NR group, five patients developed complications and one patient required laparotomy. Our study shows that percutaneous ultrasound-guided renal biopsy can be safely and successfully performed entirely by nephrologists without outside assistance. In our series, nephrologists who performed solely took fewer attempts, had better yield and fewer complications when compared to biopsies performed with radiologist’s assistance. More and more nephrologists should take up this simple yet vital procedure.

## Introduction

Percutaneous renal biopsy is an important procedure for many patients with renal disease. It was first reported in 1934 by Ball and became a routine procedure later.[1] The kidney was originally localized by correlating plain radiographs with anatomical landmarks.[2] Plain radiographs, image intensification fluoroscopy with pyelography, radioisotope scanning, retrograde pyelography, static or real-time ultrasound and computed tomography have all been used to localize the kidney for subsequent biopsy. However, it was not always possible to obtain the biopsy specimen and the procedure was not free of morbidity. The use of ultrasonography has made renal biopsies safer and easier. This was compounded by the invention of automated core biopsy devices which have made performing biopsies less taxing.

Nowadays, renal biopsy is carried out by interventional radiologists or by nephrologists with radiologist’s assistance. Quite often, fitting an emergency renal biopsy into the radiologist’s busy schedule is difficult. Another area of concern is when post biopsy complications arise and the radiologist is not available for performing a repeat scan on the patient. Also, the ability to guide the needle into the exact biopsy location depends on the radiologist’s competence. As more and more nephrologists are taking up renal ultrasound, biopsies can also be performed without radiologist’s assistance.

Renal ultrasonography by the nephrologists was popularized by O’Neill in the 1990s. He reported that diagnostic information and quick initiation of therapy was easily established when a nephrologist was involved.[3] Recent data from an academic center in USA have shown a significant reduction of the time required to perform a renal ultrasound on an outpatient basis from a mean 46.5 ± 2.4 to 4.7 ± 0. 7 days when the procedure was performed by a nephrologist.[4] Similar delays for the performance of a renal ultrasound on an outpatient basis are usual in the hospitals of Thessaloniki.[5] Nephrologists should be trained adequately in radiology laboratories with ultrasound imaging technique, where they can practice ultrasonography of kidneys or ultrasound-guided renal biopsies. The key for successful interpretation of renal ultrasonography is the correlation with the patient’s clinical problems. Therefore, a nephrologist is best suited to interpret the findings of renal ultrasonography.[6,7]

We have been performing and interpreting renal ulrasonograms over the past year. Because of the practical difficulties encountered in scheduling biopsies, we decided to perform real-time ultrasound-guided renal biopsies entirely by ourselves. Our aim was to assess the safety and effectiveness of such biopsies.

## Materials and Methods

We started doing percutaneous renal biopsies without radiologist’s assistance from March 2009. All the biopsies done at our two hospitals from March till December 2009 were included in our study. All patients underwent a set list of investigations including hemoglobin, platelets, bleeding time, clotting time and prothrombin time prior to biopsy. Abnormalities in these were corrected prior to biopsy. Renal biopsy was not done in the scheduled patients with hypertension (BP > 140/90 mm Hg) till the blood pressure (BP) was brought down to the normotensive range. Both the ultrasound and the renal biopsy were performed on all the patients by the same nephrologist using Toshiba Xario or GE Logiq ultrasound machines. Automated biopsy guns (Biopty Bard18 and 20 G) were used. Biopsy was done in the prone position with patients lying with the abdomen on a pillow. In case of renal allografts, renal biopsy was done in the supine position.

Informed consent was obtained from all the patients. The patient’s skin surface was cleansed and draped. Then 3.5 MHz transducer was used to localize the lower pole of the native kidney or the upper pole in case of the renal allograft. The distance to the biopsy point from the skin surface was assessed and the skin surface was marked at the expected needle entry point. The skin, subcutaneous, and peri-renal tissues were infiltrated with local anesthetic using ultrasonic guidance, ensuring adequate local anesthesia along the intended biopsy pathway. A small incision was made through the weal to facilitate passage of the biopsy needle. The biopsy needle was then directed through the skin incision, and then under real-time ultrasonic guidance toward the lower pole of the kidney or the upper pole of the renal allograft. Patients were asked to hold their breath when the needle approached the kidney. Advancement of the needle was halted when the tip of the needle was seen to penetrate the renal capsule. The gun was then fired, instantaneously advancing the cannula over the stylet and obtaining a core of renal parenchyma. The sampling time was less than 1 second. Repeat passes were performed to obtain two or three adequately sized biopsy specimens, if required. After the procedure, the kidney was scanned to assess for the presence of hematoma or active bleeding. All the patients were observed in our renal intensive care unit (ICU) by trained renal nurses for a period of 4 hours post procedure. The patients were returned to the hospital ward for overnight observation if there were no complications. A second check ultrasonogram was done at 24 hours just before discharge to watch for any peri-renal bleed or hematoma which would have developed later.

We compared the data with the immediate previous 37 biopsies that were performed by nephrologists with radiologist’s assistance. Here, the radiologist guided the needle under real-time ultrasound and the nephrologists performed the biopsy. All the biopsies were performed by the same radiologist and nephrologists. Statistics was done using SPSS 16 software.

## Results

Thirty-seven patients underwent percutaneous renal biopsy over a 10-month period (N group). They were compared with a similar number of biopsies done with radiology support (NR group). The baseline characteristics of both the groups are given in Table 1.

**Table 1:** Baseline characteristics of patients and indications for renal biopsy

	Nephrologist alone	Nephrologist with radiologist assistance
Total number of patients	37	37
Age (years)	38.22 ± 17.17	44.03 ± 19.16
Male:female	26:11	25:12
Number of native kidney biopsies	33	32
Number of allograft biopsies	4	5
Indication for biopsy		
Nephrotic syndrome	8	10
Nephritic syndrome	6	3
Suspected non-diabetic renal disease in a diabetic	5	4
Subnephrotic proteinuria	4	6
Delayed recovery of ARF	4	2
Chronic kidney disease - cause not known	3	5
Renal allograft dysfunction	3	5
Macrohematuria	1	0
Henoch Schonlein purpura	1	0
Multiple myeloma with renal failure	1	1
ARF of unexplained cause	1	1

ARF = acute renal failure

The commonest indication was nephrotic syndrome. Other common indications included nephritic syndrome, renal allograft dysfunction, delayed recovery in acute renal railure and suspected non-diabetic renal disease in a patient with diabetes mellitus. The characteristics of the renal biopsy specimens and complications are given in Table 2.

**Table 2:** Characteristics of renal biopsy specimens and complications

	Nephrologist alone	Nephrologist with radiologist assistance	*P* value
Total number of attempts	64	86	0.00*
No. of tissue bits obtained	49	70	NS
No. of attempts per bit	1.22	1.22	NS
No. of attempts per person	1.72	2.32	0.001*
Average size of the bit (cm)	1.40 ± 0.47	1.19 ± 0.42	NS
No. of glomeruli	15.62 ± 5.26	13.76 ± 7.38	0.00*
No. of adequate biopsies (eight or more glomeruli)	36 (97.3)	28 (75.68)	0.00*
Complications	2 (5.4)	5 (13.51)	NS
Complications requiring interventions	0	1	NS

* Significant; NS = not significant; Figures in parenthesis are in percentage

**Figure 1:**
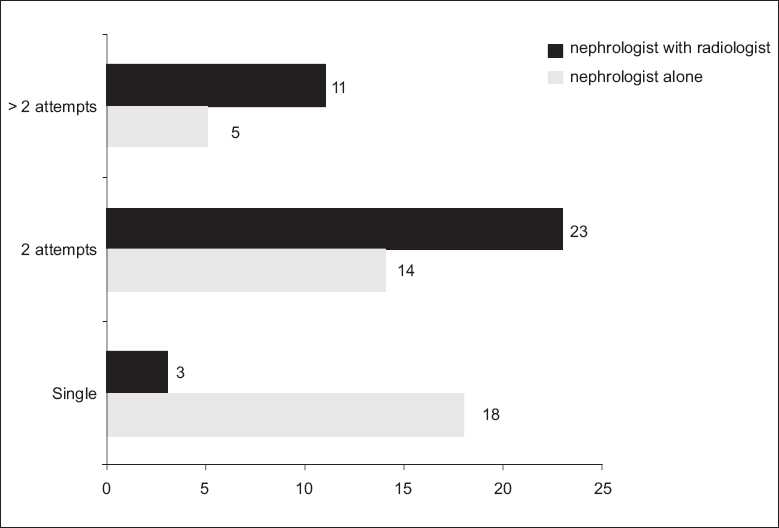
Bar diagram showing the frequency of attempts

**Figure 2:**
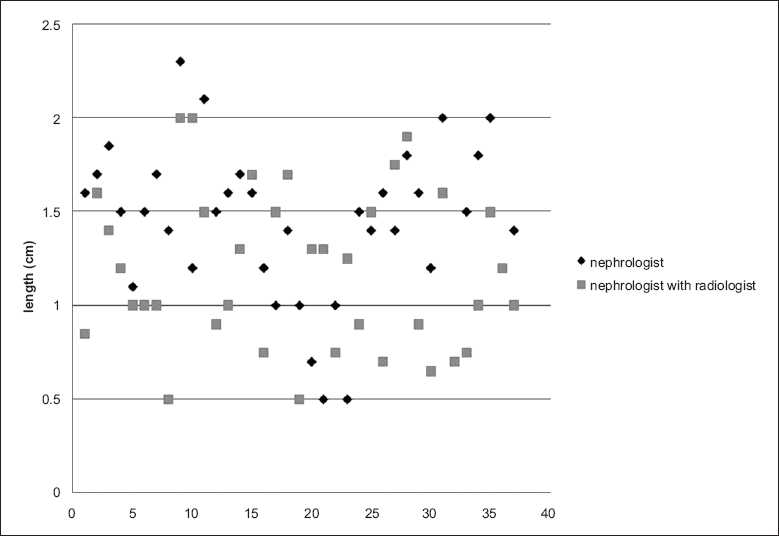
Scatter diagram showing the length of biopsy cores in various cases

There were significantly more attempts per person in the NR group [Figure 1]. Also, only 75.68% had a histopathologically adequate biopsy specimen (eight or more glomeruli) in the same group, whereas 97.3% in the N group had an adequate specimen Figure 2 shows the lengths of the biopsy cores.

**Figure 3:**
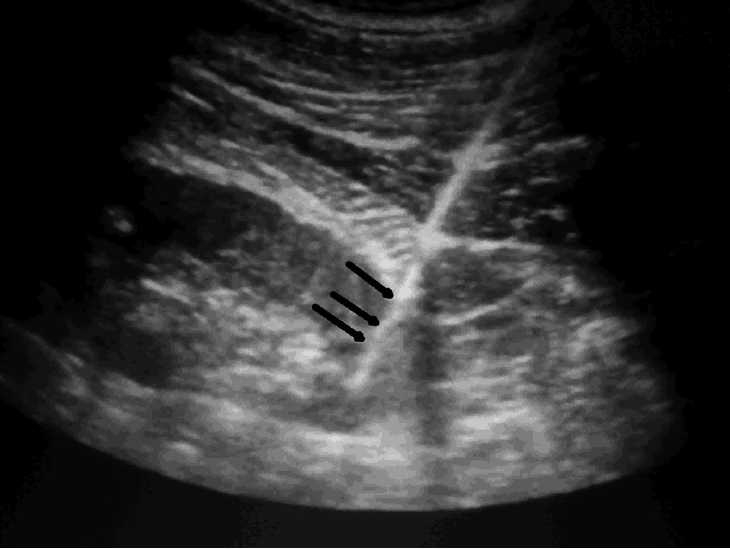
Ultrasound image showing the biopsy gun inside the lower pole of the kidney (arrows)

There were no complications in the N group except in two cases. Both had post procedural hematuria that was managed conservatively. There was no need for blood transfusion and both of them were discharged after 48 hours. No patient had peri-renal collection or hematoma on repeat ultrasonography of the abdomen at 24 hours. In the NR group, five patients developed complications. One patient had to undergo exploratory laparotomy and removal of the peri-nephric hematoma. Figure 3 shows the appearance on real time ultrasound with the biopsy gun inside the lower pole.

## Discussion

Ultrasound-guided percutaneous renal biopsy using an automated spring-loaded biopsy device has made renal biopsy safe and reliable.[8] The success rate in various ultrasound guided renal biopsy series ranges from and the adequacy varies from 88.4 to 95%.[9,10] In our series, both the adequacy and success rate was 97.29% in the N group, whereas it was 75.68 and 100%, respectively, in the NR group. The number of glomeruli per core ranged from 9 to 33.[8,11] However, all these used a bigger biopsy gun. Our yield in the N group was an average of 15.62 ± 5.26 glomeruli which was histopathologically adequate for diagnosis. In the NR group, it was 13.76 ± 7.38 glomeruli, which was slightly less than the N group and was not significant. However, the number of attempts was significantly higher in the NR group and so was the number of attempts per person. Complication rates varied from 3.36[8] to 14.3%.[12] The complication rate in our series was 5.4% in the N group and 13.51% in the NR group. In the NR group, one subject had to undergo surgical intervention to stem the bleeding.

Gupta and Balogun assessed the differences in glomerular yield and immediate procedure-related complications between radiology-performed (RP) and nephrology-performed (NP) percutaneous native kidney biopsies at their institution over a 11-month period.[13] All biopsies were done with real-time ultrasound guidance. A total of 37 native kidney biopsies were performed during the study period. Twenty-three biopsies (62%) were performed by a nephrology fellow, whereas 14 biopsies (38%) were performed by the radiologist. The mean glomerular count for RP biopsies was 15 ± 10 and for NP it was 16 ± 11 (*P* = NS). The number of passes ranged from 1 to 5 in the RP group with a total of 33 passes in 14 patients (mean 2.36 passes/patient). Passes ranged from 1 to 6 in the NP group with 57 passes in 23 patients (mean 2.48 passes/patient). Mean complication scores were similar in both the groups. However, severe complications were significantly less in nephrology fellow performed biopsies (*P* = 0.001). Despite similar pre-biopsy risk assessment and treatment protocols with real-time ultrasound guidance of all biopsies, these results showed no statistically significant differences in glomerular yield or overall complication scores but did demonstrate a significantly higher rate of severe complications in the RP biopsies. Our study also showed this trend. They concluded that nephrology fellows perform native kidney biopsies at a level equal to or superior to radiologists. Also, nephrologist-obtained renal biopsies yield similar numbers of glomeruli but are with fewer severe complications as compared with those of radiologists. In our series, both the number of passes was significantly less (1.77) and the glomerular count was similar.

The safety and efficacy of percutaneous biopsy of native kidneys performed entirely by nephrologists at the patient’s bedside was evaluated in 101 consecutive patients by Nass and O’Neil. However, only the location and depth of the kidney was assessed using ultrasound, and the actual biopsy was done without direct ultrasonic guidance. They concluded that percutaneous biopsy of native kidneys can be adequately and safely performed in its entirety by nephrologists at the patient’s bedside.[11] A comparison between their and our study is given in Table 3. In our study we experienced that the time to proceed with renal biopsy was much less when we were doing the guidance compared to previous times when we used to depend on the radiologist.

**Table 3:** Comparison between our study and the study by O’Neil and Nass

	Nass K, O’Neil^11^	Our study
No. of patients	101	37
Biopsy gun used (gauge)	15	18, 20
No of successful biopsies	99	36
Average no. of glomeruli	33	15.2 + 5.26
No. of attempts	Four or less in 80%	Two or less in 86.5%
Symptomatic hematuria	3	1
Asymptomatic hematuria	2	1

As mentioned earlier, nephrologists are the persons who are most suited to interpret and perform ultrasound-guided procedures on the kidney. Despite the importance of ultrasonography in the practice of nephrology, incorporation of training in diagnostic ultrasound into nephrology training programs is limited.[14] Nephrology training curriculum showed included adequate training in renal imaging and imaging-dependent interventions.

## Conclusion

Our study reaffirms the fact that real-time USG guided renal biopsy can be performed easily and safely by nephrologists without assistance. Also, NP biopsies have better yield and fewer complications when compared to biopsies performed with radiologist’s assistance. More and more nephrologists should take up this simple yet vital and rewarding procedure.
